# *Ex vivo* assessment and *in vivo* validation of non-invasive stent monitoring techniques based on microwave spectrometry

**DOI:** 10.1038/s41598-018-33254-9

**Published:** 2018-10-04

**Authors:** Carolina Gálvez-Montón, Gianluca Arauz-Garofalo, Oriol Rodriguez-Leor, Carolina Soler-Botija, Susana Amorós García de Valdecasas, Flavio David Gerez-Britos, Antoni Bayes-Genis, Juan Manuel O’Callaghan, Ferran Macià, Javier Tejada

**Affiliations:** 1 ICREC Research Program, Fundació Institut d’Investigació en Ciències de la Salut Germans Trias i Pujol, Barcelona, Spain; 2 CIBER Cardiovascular, Instituto de Salud Carlos III, Madrid, Spain; 3 Grup de Magnetisme, Departament de Física de la Matèria Condensada, Universitat de Barcelona, Barcelona, Spain; 4 Servei de Cardiologia, Hospital Universitari Germans Trias i Pujol, Badalona, Spain; 5 Departament de Medicina, Universitat Autònoma de Barcelona, Barcelona, Spain; 6 CommSensLab, Universitat Politècnica de Catalunya, Barcelona, Spain

## Abstract

Some conditions are well known to be directly associated with stent failure, including in-stent re-occlusion and stent fracture. Currently, identification of these high-risk conditions requires invasive and complex procedures. This study aims to assess microwave spectrometry (MWS) for monitoring stents non-invasively. Preliminary *ex vivo* data are presented to move to *in vivo* validation. Fifteen mice were assigned to receive subcutaneous stent implantations (*n* = 10) or sham operations (*n* = 5). MWS measurements were carried out at 0, 2, 4, 7, 14, 22, and 29 days of follow-up. Additionally, 5 stented animals were summited to micro-CT analyses at the same time points. At 29 days, 3 animals were included into a stent fracture subgroup and underwent a last MWS and micro-CT analysis. MWS was able to identify stent position and in-stent stenosis over time, also discerning significant differences from baseline measures (P < 0.001). Moreover, MWS identified fractured vs. non-fractured stents *in vivo*. Taken together, MWS emerges as a non-invasive, non-ionizing alternative for stent monitoring. MWS analysis clearly distinguished between in-stent stenosis and stent fracture phenomena.

## Introduction

Coronary artery disease is the leading cause of death worldwide. It contributes to more than 7 million deaths annually^1^. Percutaneous coronary intervention with stenting is the most widely performed procedure for the treatment of symptomatic coronary artery disease^2^. Although drug-eluting stents have minimized the limitations of bare-metal stents, serious concerns remain about late complications, such as in-stent restenosis and late stent thrombosis^3^. Some conditions are known to be directly associated with stent failure, including in-stent neointimal proliferation, late malapposition related to positive vascular remodeling, stent fracture, and in-stent neoatherosclerosis^4^. Pre-clinical identification of these high-risk conditions requires invasive, complex procedures, such as coronary angiography, intravascular ultrasound (IVUS), optical coherence tomography (OCT), or multislice computed tomography^5,6^. Several high frequency-based alternatives for monitoring stents have been investigated^7–9^, but require complex solutions such as prior incorporation of electronic chips to the stent.

Recent studies have assessed the prospect of a new, non-invasive, non-ionizing stent monitoring method based on microwave spectrometry (MWS)^10–12^. In contrast to the other approaches, MWS does not require prior stent modifications and should be valid with all currently available metallic stents.

In this context, MWS -defined as a measurement of the frequency response of the sample to microwave electromagnetic fields- is a potentially non-invasive and non-ionizing stent monitoring technique. The principle of the MWS technique relies on the interaction between an electromagnetic wave and an object with high conductivity, such as a metallic stent. Similar to a tuning fork that resonates at particular sound frequencies, the electrons from the stent alloy establish standing waves of current that resonate at microwave frequencies^10,11^. From the characteristic resonance frequencies of the stent, we can obtain information about the stent status. In this work we have set up a new approach for detection of stent structural failure and in-stent lipid occlusion, and thus, for the first time, we assessed *in vivo* MWS for stent monitoring.

## Methods

A detailed description is provided in the Supplementary Material.

### *In vitro* stent testing

The *in vitro* methods of the present work comprise three tests in which a commercial coronary stent (see Supplementary Table 1 for details) is submitted to different processes to emulate common stent-related complications: stent fracture, diametrical stent collapse (recoil), and neointimal atherosclerotic transformation inside a stent (in-stent lipid occlusion).

#### Stent fracture test

Once expanded, the stent length (*ℓ*_AB_) was measured using an IP67 digital caliper (Vogel Germany GmbH & Co., Kevelaer, Nordrhein-Westfalen, Germany) and its *A*(*f*, *φ*) chart was acquired. Next, we split the stent into two segments by cutting the weld linking two adjacent crowns with customized scissors, which led to a grade III fracture^12^. The lengths (*ℓ*_A_ and *ℓ*_B_) of the resulting segments were also measured. The *A*(*f*, *φ*) chart for the fractured stent was acquired by placing both segments 1 mm apart on the sample holder.

#### Stent recoil test

To simulate recoil, we smoothly changed the stent diameter along a wide range of values. The variations were progressive and uniform along the entire length of the stent. The pressure of the balloon catheter, *p*, was increased in a stepwise manner. At each stage, the *A*(*f*, *φ*) chart and diameter of the stent, *d*, were measured using an IP65 digital micrometer (Vogel Germany GmbH & Co., Kevelaer, Nordrhein-Westfalen, Germany).

#### In-stent lipid occlusion test

Once expansion of the stent was completed, it was subjected to progressive cholesterol deposition to simulate worsening in-stent lipid occlusion. The cholesterol deposition process was carried out by spraying the stent with a cholesterol-saturated (94%, Sigma-Aldrich) ethanol (96%, Panreac) solution.

### Murine animal model and *in vivo* micro-CT analysis

Animal studies were approved by the Germans Trias i Pujol Research Institute Animal Experimentation Ethics Committee (B9900005) and the Generalitat de Catalunya government (DARP: 8688),  and complied with the Guide for the Care and Use of Laboratory Animals (NIH Publication No. 8023, revised 1996). Eleven bare-metal stents (Stent Architect, 4.00 mm diameter, 19 mm length; iVascular, S.L.U) were used. Ten were implanted in mice, and one was characterized in open-air conditions, as a reference. Fifteen C57/Bl6 female mice (18 ± 1 g; 10 weeks old; Harlan Laboratories, SA.) were randomly distributed into: (1) experimental group (n = 10) with stent implantation, and (2) sham group (n = 5) with simulated stent implantation. Briefly, animals were anesthetized with a mixture of O2/isoflurane. Buprenorphine (0.1–0.5 mg/kg) was administered subcutaneously, as intraoperative analgesia. Next, after a paravertebral incision, a subcutaneous tunneling was performed to place the stent at 7.0 ± 0.5 atm. Finally, the surgical wound was closed with cyanocryolate glue (3M TM Vetbond TM Tissue Adhesive, 3M), and animals were recovered.

**Figure 1:**
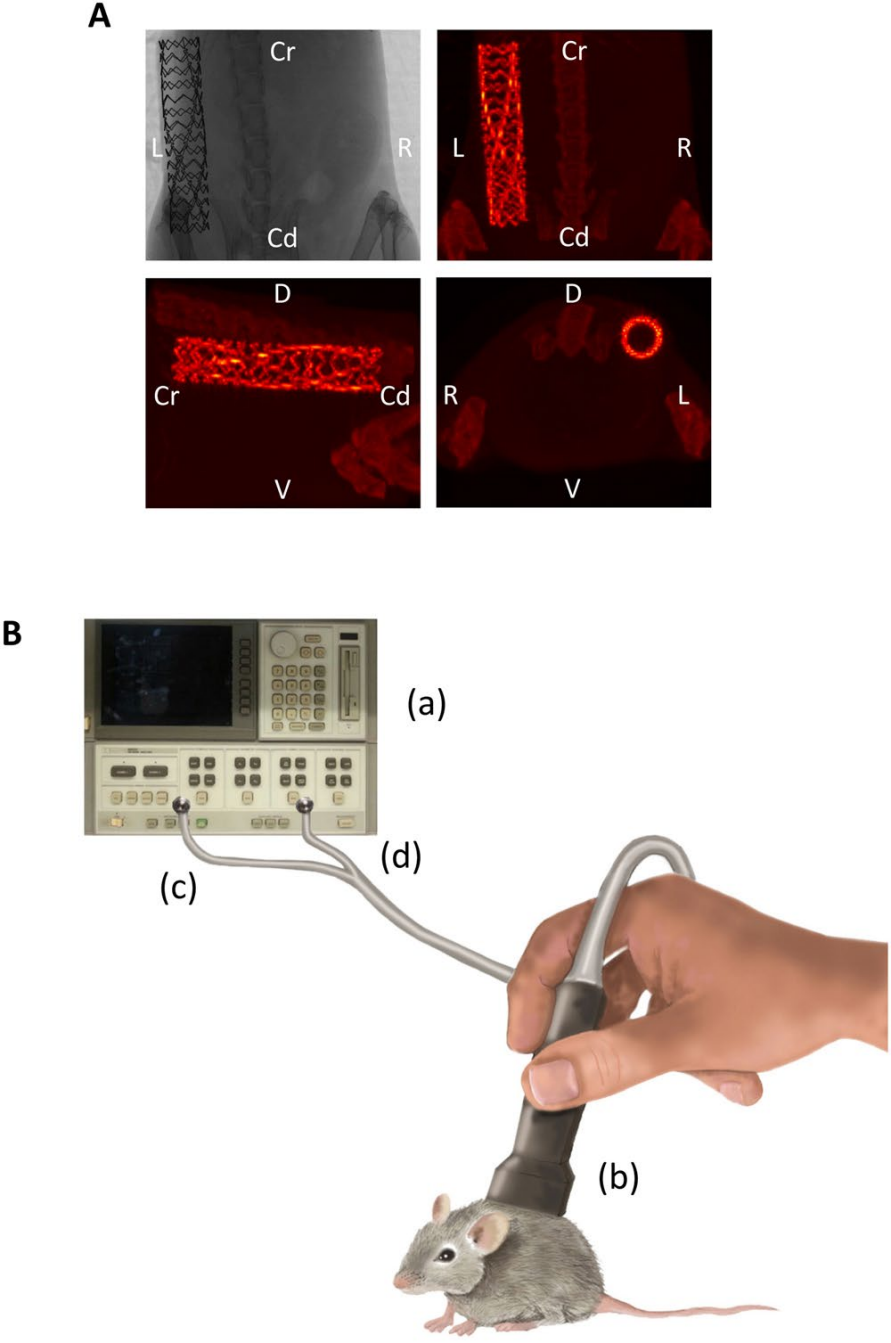
Sketch of the MWS setup. (**A**) Representative micro-CT images display the location of the stent. (**B**) The network analyzer (a) sends an electromagnetic stimulus to the MWS probe (b) through a coaxial feedline (c). The generated microwave signal travels through mouse tissue, until it interacts with the stent. The emerging signal is collected by the MWS probe and sent back to the network analyzer via a second feedline (d). The figure was designed and hand-drawn by Carolina Gálvez-Montón. Cr = Cranial; Cd = Caudal; L = Left; R = Right; D = Dorsal; V = Ventral.

Baseline MWS measurements were carried out after stent grafting (Day 0). Further measurements were done at 2, 4, 7, 14, 22, and 29 days. Additionally, 5 animals were submitted to micro-CT (Skyscan 1076 high resolution *in vivo* scanner, Kontich) analysis at the same time points, to detect deformations and stent breakage over time (Fig. 1A). Imaging was performed with gating for respiratory motion, and scan parameters were optimized to reduce scanning time and radiation dose. The radiation scan was applied with 45 kV source voltage and 250 µA, 18 µm isotopic resolution, one projection image per 0° gantry rotation, rotation range 180°, and a field of view covering the thorax region. A titanium filter was used. The total standard acquisition time was 15 min.

At the end of the study, 3 of these 5 animals were submitted to stent fracture. Fractures were produced by making a transverse cut in the stent, mimicking a type III fracture according to Allie-Jaff classification^12^. Cuts were made at different sites on the 3 stents (1/2, 1/4, and 1/5 of the stent length). Then, final MWS scan and micro-CT analyses were performed. Finally, animals were sacrificed with an overdose of anesthesia and stents were removed and photographed.

### *In vivo* microwave spectrometry setup

It consisted of a two-port probe connected to a vector network analyzer (E8361A, Keysight Technologies) via coaxial feed lines (3GW40-0TD01D02048.0, W. L. Gore & Associates) (Fig. 1B). The probe was used to launch a microwave electromagnetic excitation onto the stent and to capture its response. To ensure that the microwave electromagnetic fields did not affect the biological tissues and conformed to the restrictions set in^13^, we performed a Finite Element Method (FEM) simulation using commercial software (ANSYS HFSS). The parameters of the biological tissues used in the simulation (relative permittivity ${\epsilon }_{r}$ and conductivity *σ*) where measured using a commercial device (85070E dielectric probe kit, Keysight). The simulation was run on a 5 mm thick slab of material with ${\epsilon }_{r}=52$ and *σ* = 1 *S*/*m* and showed that, with the microwave power used in the experiments (1 mW), the Specific Absorption Rate (SAR)^13^ was negligible everywhere except in a small region near the probe with a depth under 2 mm and a perimeter of 4.1 mm. The maximum SAR in that region was 6.6 W/kg, below the 10 W/kg limitation set for occupational exposure in^13^.

The transmission spectrum (TS) was acquired with our probe in a frequency range of 0.1 to 6.0 GHz, to identify the resonance frequencies characteristic of a stent implanted in mice.

Measurements were performed with anesthetized mice lying in a prone position on a padded surface, and their backs were palpated with the MWS probe. Ultrasound transmission gel (Aquasonic 100, Parker Laboratories, Inc.) was applied on the probe head to soften the medium transitions and convey microwaves without crossing air. Each experimental mouse characterization comprised 3 measurements at different scanning zones. The first 2 zones were both with the probe centered above the stent, one parallel and the other angled at 30° with respect to the stent axis. The third zone was shifted 10 mm to the right of the paravertebral position. Sham mouse characterizations comprised the same 3 measurements.

### Statistical Analysis

Differences in MWS analyses over time were evaluated with ANOVA for repeated measures and the Greenhouse correction, when Mauchly sphericity was not achieved. Data are presented as the mean ± standard deviation, and statistical significance was defined as P < 0.05.

The datasets generated and/or analyzed during the current study are available from the corresponding author on reasonable request.

## Results

### *Ex vivo* Analysis

#### Microwave spectrometry absorbance charts

**Figure 2:**
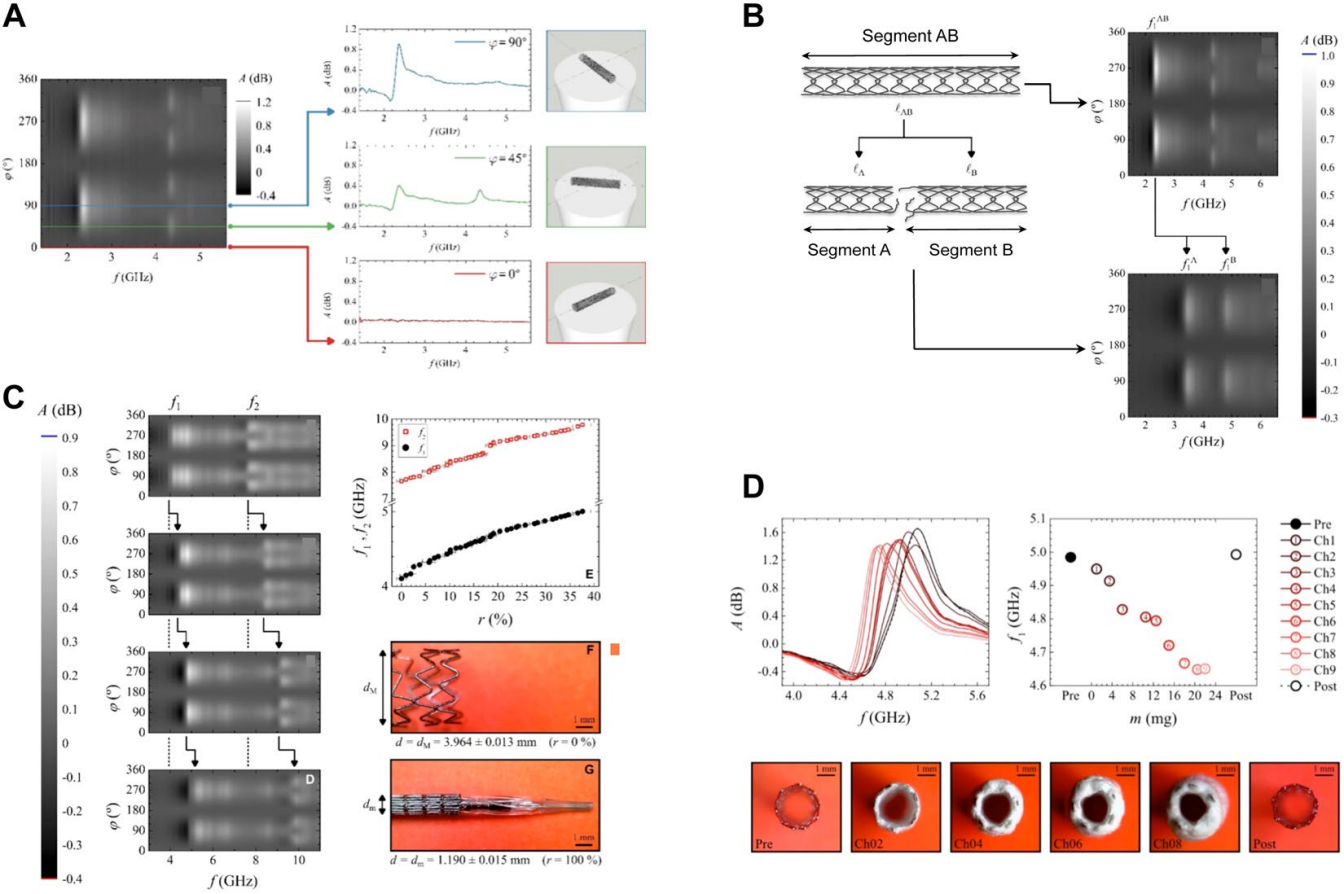
*Ex vivo* tracking stent status with MWS. (**A**) Image displaying a typical MWS stent scan. (*Left*) Absorbance chart as a function of the MW frequency and scanning angle (*A*(*f*, *φ*)) of a Medtronic Endeavor Sprint DES (30 × 2.50 mm). (*Right*) Isoangular projections of the *A*(*f*, *φ*) chart at *φ* = 90°, 45°, and 0°. (**B**) Fracture test performed on a Medtronic Endeavor Sprint stent (30 × 2.50 mm). The stent (AB) (length 30.74 ± 0.08 mm) is split into two segments (18.58 ± 0.05 mm and 12.16 ± 0.03 mm.; A and B) The original resonance (2.306 ± 0.004 GHz) (top right) splits into two resonant frequencies (3.404 ± 0.008 GHz and 4.766 ± 0.012 GHz) (bottom right). (**C**) Recoil test performed on a Medtronic Endeavor Resolute stent (15 × 3.00 mm). Absorbance chart for the stent at its maximum diameter (minimum recoil) (*d*_M_ = 3.964 ± 0.013 mm, *r* = 0.0 ± 0.1%) (*Left*) and absorbance charts with decreasing diameter (*d* = 3.624 ± 0.013 mm; 3.333 ± 0.015 mm; and 2.92 ± 0.03 mm). (*Top Right*) Graphical evolution of the first two resonant frequencies, *f*_1_ and *f*_2_, for decreasing diameter (increasing recoil degree). (*Bottom Right*) Representative images showing the maximum and minimum diameters (*d*_M_ and *d*_m_) used as references to define the recoil degree. (**D**) In-stent neoatherosclerosis test performed on a Medtronic Driver Sprint BMS (12 × 2.75 mm). (*Top Left*) Stent isoangular spectra at *φ* = 90° at each stage reached during the test, showing the shape of its fundamental resonance and a global downshift. (*Top Right*) Evolution of the fundamental resonant frequency with an increasing cholesterol depot, *f*_1_(*m*). (*Bottom*) Axial sequential imaging illustrates the evolution of the cholesterol crust (from left to right: *m* = 0.0 ± 0.5 mg, 3.5 ± 0.5 mg, 10.5 ± 0.5 mg, 15.0 ± 0.5 mg, 20.5 ± 0.5 mg, and 0.0 ± 0.5 mg). *A = *Absorbance; *d = *Diameter; *d*_M_ = Maximum diameter; *d*_m_ = Minimum diameter; dB = Decibels; *f* = Microwave frequency; GHz = Gigahertz; *φ* = Angle.

Figure 2A presents the *A*(*f*, *φ*) chart for the stent used in fracture test to illustrate a typical MWS characterization. Each isoangular projection in this chart (horizontal line) represents the absorbance spectrum for a given *φ* value. Figure 2A shows the isoangular profiles at *φ* = 90°, 45°, and 0°. Note how the isoangular projection at *φ* = 90°shows a resonance (maximum absorbance), the precise frequency value of which can be extracted by applying fitting tools (*f*_1_ = 2.306 ± 0.004 GHz). This resonance is observed again with the isoangular projection at *φ* = 45°, but with a reduced amplitude. In this same *φ* = 45° isoangular projection, we detected an *f*_2_ of 4.317 ± 0.004 GHz, which was not present at *φ* = 90°. Finally, we observed that these two resonances eventually disappeared at *φ* = 0°.

#### Stent fracture test

Figure 2B summarizes the fracture test by comparing the two *A*(*f*, *φ*) charts taken before and after applying the cut. The fundamental resonance of the original stent (segment AB) is easily spotted in this chart (*f*_1_^AB^ = 2.306 ± 0.004 GHz). The *A*(*f*, *φ*) chart of segments A and B placed together as described above is also included. As notable result, there is no trace of the original *f*_1_^AB^ in this new chart. Instead, a pair of new resonances (*f*_1_^A^ = 3.404 ± 0.008 GHz and *f*_1_^B^ = 4.766 ± 0.012 GHz) appear at higher frequencies. The lengths of the intact segment AB and segments A and B resulting from the cut are 30.74 ± 0.08 mm, 18.58 ± 0.05 mm, and 12.16 ± 0.03 mm, respectively.

#### Stent recoil test

Using non-invasive MWS we were able to identify a gradual up-shift of stent resonances as the stent diameter was reduced. After the recoil test carried out on the MER_1500_300 stent, MWS measurements displayed the *A*(*f*, *φ*) chart for the stent in the absence of recoil (*r* = 0.0 ± 0.1%), and the *A*(*f*, *φ*) charts for increasing recoil degrees (*r* = 12.3 ± 1.0%, 22.7 ± 1.1%, and 37.6 ± 1.7%, respectively). Figure 2C provides the full view of the process by plotting the evolution of *f*_1_ and *f*_2_ as *r* increases. Finally, the micropictures shown in Fig. 2C show the stent at the extreme diameter values, *d*_M_ and *d*_m_, used to define the recoil degree (see Supplementary Material).

#### In-stent lipid occlusion test

Figure 2D shows how MWS is able to detect the amount of cholesterol deposited on the stent by monitoring the absorbance resonance at *φ* = 90°. There is an important frequency downshift as cholesterol mass accumulates on the stent, with a maximum downshift of 6.70 ± 0.14%. Figure 2D (Bottom) presents a sequence of six axial micropictures showing the progress of the cholesterol crust.

### *In vivo* validation

#### Stent detection with MWS

**Figure 3:**
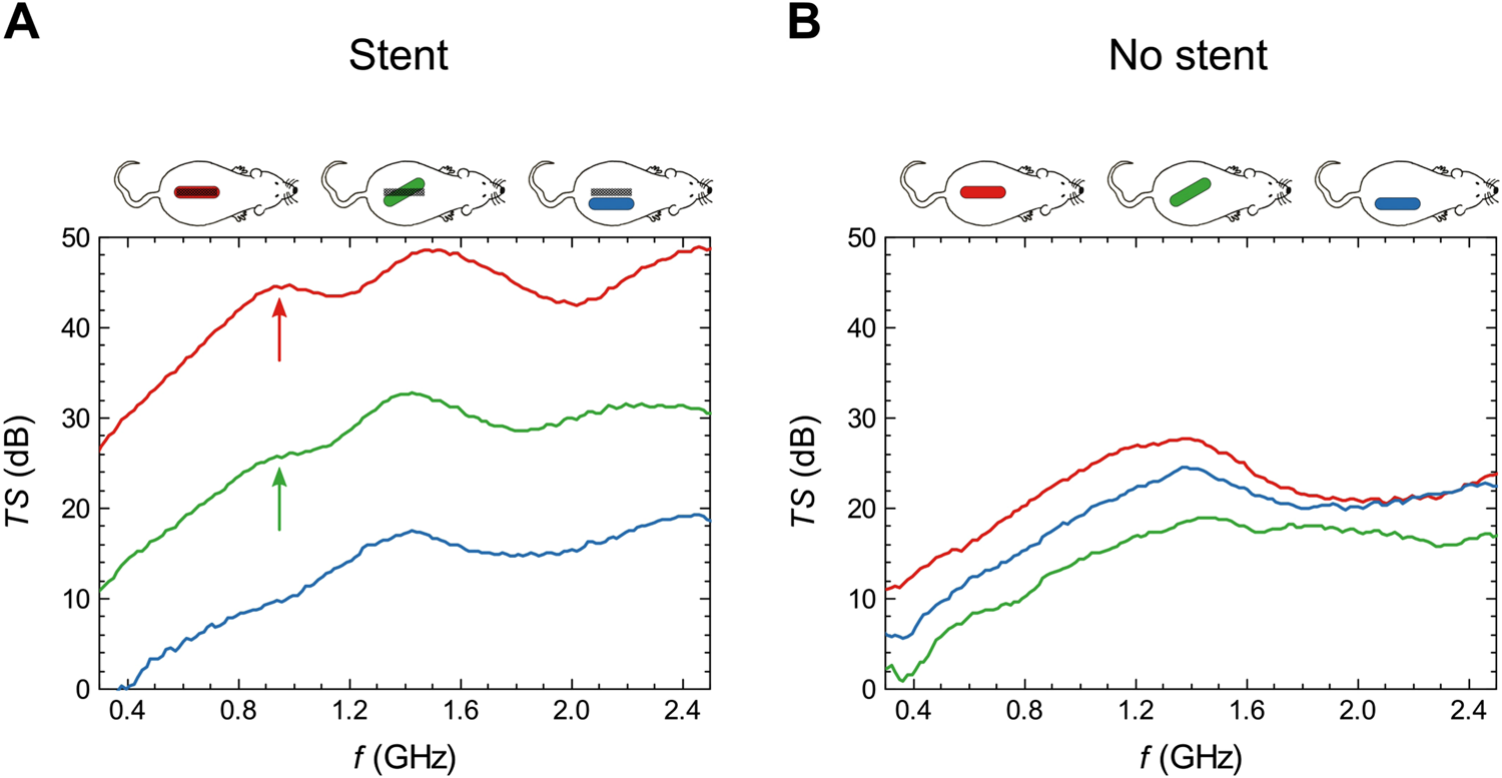
*In vivo* detection of the stent by MWS. Transmission spectra (TS) acquired in animals with implanted stents (**A**) and with sham implantations (**B**) on the 29th follow-up day. Different colors represent distinct scanning positions for the microwave spectrometry (MWS) probe, with respect to the implantation zone (experimental group) or the simulated stent implantation zone (sham group). Red and green spectra of stented animals show a distinctive, shoulder-shaped signal, which is not present in either the blue spectrum (red and green arrows) or the three spectra measured in the sham mouse. TS = Transmission Spectrum; dB = Decibels *f* = Microwave frequency, GHz = Gigahertzs; MWS = Microwave Spectometry.

Comparisons of the MWS signals acquired from experimental and sham animals allowed us to establish the stent presence and location. Figure 3 illustrates the stent presence indicators provided by MWS performed on the 29th follow-up day. The red, green and blue areas on the mice contours in Fig. 3 indicate the position of the probe. Note that maximum signal is obtained when the probe is aligned with the stent and directly above it. The transmission spectrum (TS) obtained in experimental animals was compared to that acquired in sham animals. The spectra acquired from the stented animals showed remarkably larger signals than those acquired from the sham animals, even when the spectra from stented animals were acquired angling the probe (green curve). Furthermore, the stented animals displayed a resonance frequency that produced a shoulder-shaped feature in the spectra at *f* = 0.82 ± 0.10 GHz (red and green arrows), which was not present in either the blue spectrum or the 3 spectra measured in sham mice. Figure 3 shows representative data measured on a single day; analogous results were obtained for all animals during the entire monitoring period. All mice from the experimental group exhibited a characteristic shoulder-shaped resonance each day they were monitored, and no mice from the sham group ever displayed that feature.

#### In-stent stenosis

After performing the MWS measurements, we normalized all resonance frequencies for the experimental group (Table 1) to the resonance value of the first day (Day 0):

$${\rm{\Delta }}{f}_{mice}=\frac{f-{f}_{0}}{{f}_{0}}$$

**Table 1:** Resonance frequencies acquired with microwave spectrometry in mice with implanted stents.

Animal	Day 0	Day 2	Day 4	Day 7	Day 14	Day 22	Day29
M01	0.80 ± 0.20	0.76 ± 0.16	0.79 ± 0.08	0.76 ± 0.14	0.83 ± 0.09	0.79 ± 0.09	0.81 ± 0.09
M02	0.70 ± 0.08	0.79 ± 0.15	0.75 ± 0.09	0.75 ± 0.11	0.72 ± 0.09	0.76 ± 0.08	0.80 ± 0.10
M03	0.67 ± 0.11	0.76 ± 0.12	0.78 ± 0.10	0.79 ± 0.10	0.78 ± 0.12	0.76 ± 0.09	0.78 ± 0.09
M04	0.97 ± 0.10	0.76 ± 0.12	0.81 ± 0.10	0.78 ± 0.10	0.78 ± 0.08	0.78 ± 0.09	0.83 ± 0.12
M05	0.65 ± 0.07	0.81 ± 0.13	0.79 ± 0.09	0.83 ± 0.09	0.82 ± 0.10	0.76 ± 0.09	0.80 ± 0.11
M06	0.72 ± 0.18	0.76 ± 0.10	0.79 ± 0.11	0.77 ± 0.07	0.78 ± 0.11	0.84 ± 0.10	0.82 ± 0.10
M07	0.65 ± 0.07	0.68 ± 0.19	0.79 ± 0.13	0.74 ± 0.10	0.84 ± 0.10	0.82 ± 0.09	0.78 ± 0.08
M08	0.68 ± 0.06	0.74 ± 0.10	0.80 ± 0.11	0.81 ± 0.08	0.79 ± 0.09	0.81 ± 0.10	0.81 ± 0.11
M09	0.70 ± 0.08	0.76 ± 0.12	0.80 ± 0.10	0.84 ± 0.10	0.78 ± 0.09	0.79 ± 0.10	0.79 ± 0.10
M10	0.64 ± 0.08	0.79 ± 0.10	0.81 ± 0.09	0.79 ± 0.10	0.76 ± 0.09	0.77 ± 0.10	0.78 ± 0.10

**Figure 4:**
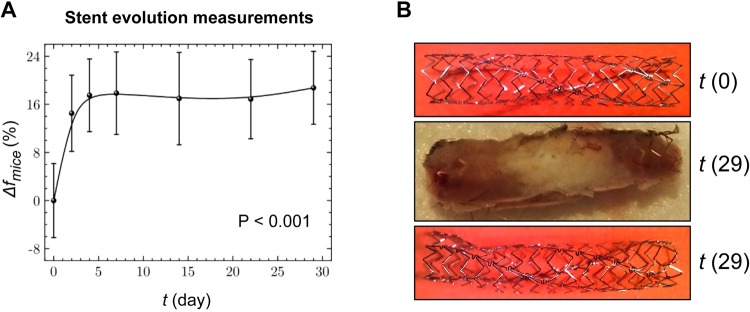
*In vivo* temporal evolution of stent resonance. (**A**) Graphic represents the relative displacement of the average resonance frequency (Δ*f*_mice_) over time, for experimental mice (n = 10). (**B**) Images of implanted stents acquired before implantation (*top*), after sacrifice at 29 days of follow-up (*middle*), and after a NaOH wash *(bottom)*. Δ*f*_mice_ = Average resonance frequency; *t* = Time.

The statistical analysis indicated that significant changes occurred over time (P < 0.001). These results suggested that in-stent stenosis could be detected based on the resonance frequency displacement Δ*f*_*mice*_ over time (Fig. 4). Note that the average resonance frequency rapidly shifted up, during the first 4 monitoring days, and then it stabilized at a relative displacement of approximately 16%.

#### Stent fracture

**Figure 5:**
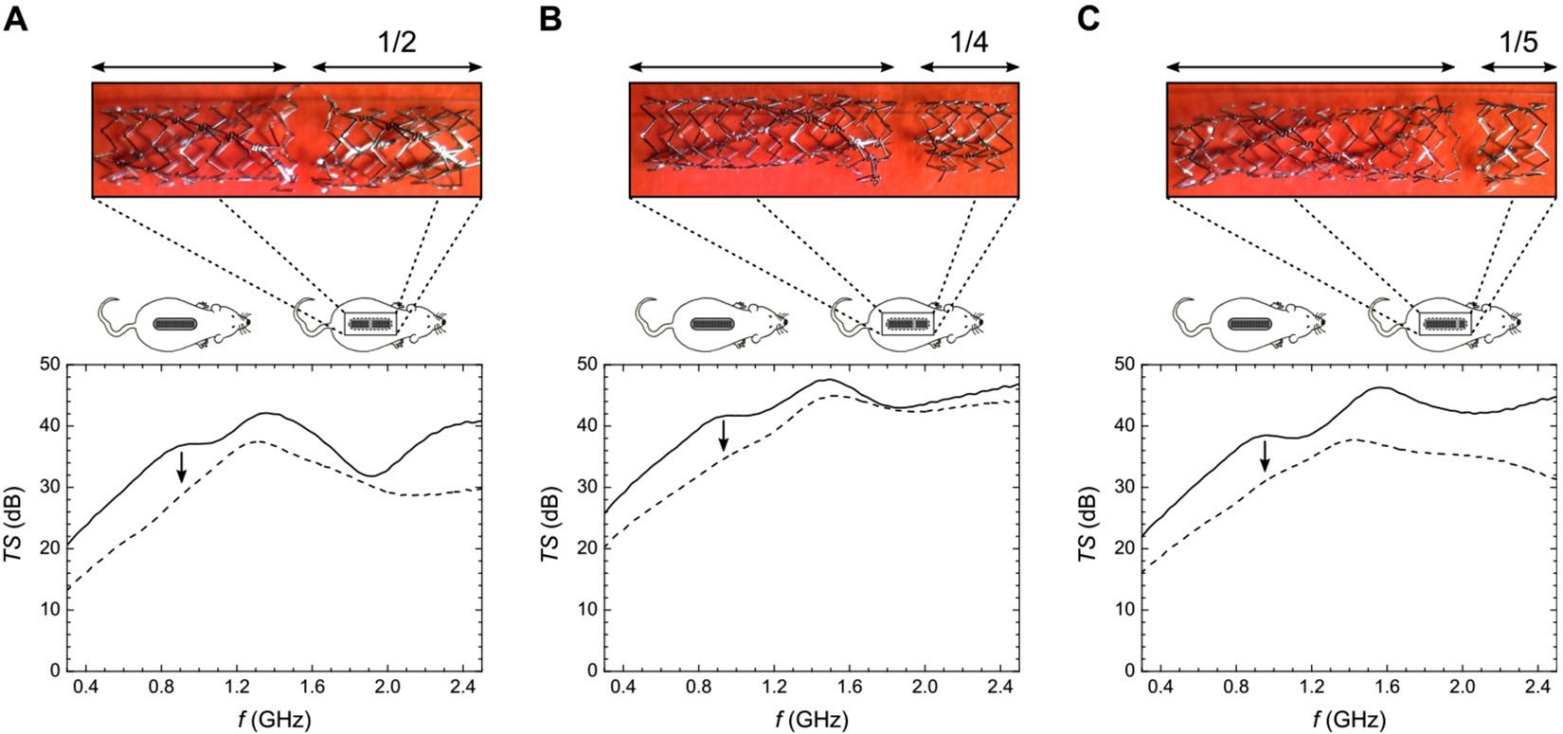
*In vivo* comparison before and after fracturing the stent. The transmission sprectrum (TS) acquired before (solid lines) and after (dashed lines) cutting the stent. In all cases, the scanning position of the microwave spectrometry (MWS) probe was just above the stent, parallel to the direction of implantation. Each stent was broken in two segments, which left fragments that were about (**A**) 1/2, (**B**) 1/4, and (**C**) 1/5 of the stent length. See corresponding sketches of mice and the associated micro-pictures. dB = Decibels; *f* = Microwave frequency, GHz = Gigahertzs; *TS* = Transmission spectrum.

To detect stent fractures with MWS, we measured the TS obtained before and after cutting the implanted stents into two segments (with short fragments of 1/2, 1/4, and 1/5 of the stent length) (*n* = 3). Figure 5 displays the results for the cuts performed at different locations along the stent shaft (see corresponding pictograms and associated micro-photographs for details). In all 3 cases, the TSs were acquired on day 29. The characteristic shoulder-shaped resonance signals found before splitting the stent, at frequencies of *f*_*S*1/2_ = 0.78 ± 0.08 GHz, *f*_*S*1/4_ = 0.81 ± 0.11 GHz, and *f*_*S*1/5_ = 0.79 ± 0.10 GHz, were lost after the stents were fractured (black arrows).

#### Quantitative analysis of the stent resonance frequency

The resonant frequency for the stents implanted in mice (*f*_mice_) is consistent with that of the stent measured in air (*f*_air_) and the relative permittivity measurements of the tissue where the stents are implanted (*ε*_mice_). Since the *in vivo* and open-air wavelengths at resonance are equal, we can extrapolate the open-air resonance frequency with *in vivo* data:

$${f}_{air}=\sqrt{{\varepsilon }_{mice}}\cdot {f}_{mice}$$

Since *f*_*mice*_ = 0.77 GHz and *ε*_mice_ is about 47 at that frequency (see Supplementary Fig. 2), the open-air resonance (*f*_air_) should be 5.28 GHz. This compares well with the measured value of *f*_air_ (5.07 GHz) and the error margins in the permittivity measurements.

## Discussion

Late stent failure represents an unresolved issue in interventional cardiology. To date, after stent implantation, coronary stent evaluation requires repeated coronary angiography. Other invasive coronary imaging techniques, such as IVUS or OCT, offer information about the stent, the vessel lumen, the vessel wall, and the plaque burden^14^. Nevertheless, IVUS and OCT are of limited use, because both techniques are invasive, expensive, and require hospital admission and a trained physician. A non-invasive methodology that could be implemented by non-specialized physicians to stratify the risk of patients after stent implantation would avoid these drawbacks.

In this context, the present study shows that MWS provides distinctive indicators for a variety of different changes in stent structure and environment *ex vivo*. On the one hand, MWS reveals stent fracture as a split-and-up-shift of the stent resonant frequency. On the second hand, stent recoil is shown as a gradual up-shift of the stent resonant frequency. The higher the diametrical shrinkage of the stent is, the larger the up-shift will be. Finally, MWS reveals cholesterol presence around stent as a downshift of its resonant frequency. A larger frequency shift corresponds to a higher cholesterol mass deposited on the stent. We expect this dependence to be even more sensitive in an *in vivo* measurement, where the ratio of permittivities of the materials involved (blood vs. cholesterol) would be higher than the one in our experiment (cholesterol vs. air). These *ex vivo* pre-clinical findings suggests us that this technology has the potential to monitor common concerns associated with coronary stents over the long-term, which are changes in stent geometry (stent fracture and recoil) and in-stent re-occlusion (in-stent restenosis or in-stent lipid occlusion).

In our *in vivo* validation, comparison of TS spectra between experimental and sham groups showed that MWS provided two indicators of stent presence. First, the level of the TS spectra was enhanced when the probe was accurately aligned with the stent (compare red and blue solid lines in Fig. 3A); this suggested the possibility of using MWS for determining stent position. Second, the presence of the stent produced a characteristic shoulder-shaped resonance at *f* = 0.82 ± 0.10 GHz (see red arrow in Fig. 3A). Note that the TS peaks at around 1.4 GHz that appeared in all experimental conditions were related to the probe design and had no significance in stent monitoring.

We used the MWS formal framework^10^ and independent permittivity measurements^11,14^ to check the agreement between open-air and *in vivo* resonance frequencies (${f}_{air}=\sqrt{{\varepsilon }_{mice}}\cdot {f}_{mice}$). This fact confirmed that the shoulder-shaped resonance in the *in vivo* measurements (Fig. 3A) was actually due to the presence of the stent.

Late-acquired stent malapposition or stent recoil is a well-recognized problem in interventional cardiology because it may give rise to late stent thrombosis, and the subsequent need for prolonging dual antiplatelet therapy. The incidence of late-acquired stent malapposition was reported to be as high as 25% in patients with acute myocardial infarction^12,15^. In this context, we also evaluated *in vivo* the in-stent stenosis with the MWS system. The evolution of the average resonance frequency showed that stents experienced notable alterations during the first 4 days after implantation, and then eventually stabilized on the fifth day. It is well known that stent resonance frequencies strongly depend on the dielectric permittivity of the surrounding medium^10,11,14^. This physical property of matter can be understood as the opposition encountered by an electric field, when it forms within a medium, such as a biological tissue. Because different tissues present distinct characteristic dielectric permittivity profiles^16^, the upward shift in the average resonance frequency we observed (Fig. 4A) could be assigned to tissue remodeling around the implanted stent (interested readers can refer to the Supplementary Material for an extended argumentation). Mouse post-mortem autopsies showed that the interstitial fluid in the stent lumen just after subcutaneous stent implant was gradually replaced by fibrotic tissue (Fig. 4B). This fibrotic phenomenon was also previously described in bare-metal stents that were subcutaneously implanted in rats^17^. Moreover, our implanted stents had adhered to the surrounding tissues, and those tissues penetrated into the stent and caused tissue proliferation mimicking in-stent stenosis. As we have demonstrated, the MWS system indicated in-stent fibrotic formations and depositions that took place during the early days of follow-up in all experimental animals. Thus, MWS might offer a new option of monitoring to discern which patients are at high risk of in-stent stenosis.

Stent fracture is a late complication of stent implantation, but its true incidence remains unknown. However, rates up to 29% have been reported. Patients with stent fractures may remain asymptomatic, but they may present acute coronary syndrome, stent thrombosis, or recurrent angina due to clinical restenosis. Overall, 70% to 80% of patients with a stent fracture will present in-stent restenosis or stent thrombosis^18,19^. Using MWS we were able to identify changes exhibited by the *A*(*f*, *φ*) chart after fracturing the stent, specifically split in the original resonance producing two new higher frequencies. Furthermore, we successfully showed that *f*_1_^A^ is unequivocally due to segment A just by measuring its *A*(*f*, *φ*) separately. We also checked that *f*_1_^B^ was unequivocally due to segment B. Additionally, since the fundamental mechanism behind this split-and-shift-up behavior is well understood, the quantitative evaluation of grade III (and even grade IV) stent fractures is possible^11^.

Taken together, our results have opened up a new paradigm in stent monitoring. Although further studies in large animal models are needed to validate the present results, this study showed that MWS methodology could allow non-invasive, non-ionizing coronary stent monitoring for diagnosing different conditions related to stent failure at a pre-clinical stage.

### Study limitations

The present study described a new methodology for monitoring the status of implanted stents. With MWS analysis, we detected the presence of the stent, in-stent restenosis, and type III stent fractures. Further studies are needed to discern materials of different natures, such lipidic, thrombotic, and fibrin deposits. Moreover, different types of stent fractures should be evaluated.

## Conclusions

This study described a new MWS system, which offers, for the first time, the potential of detecting the presence of implanted stents in mice and monitoring stent restenosis and fractures. Moreover, this novel, probe-based, non-invasive, non-ionizing methodology would not require sedation, patient hospitalization, or specialized physicians. This data represented a proof-of-principle for the MWS approach, a valuable step before undertaking clinical translation.

## Electronic supplementary material

Supplementary Material
