# Supplementary material for: Ex vivo assessment and in vivo validation of non-invasive stent monitoring techniques based on microwave spectrometry
Source: Sci Rep. 2018 Oct 4;8:14808. doi: 10.1038/s41598-018-33254-9 (PMC6172211; doi:10.1038/s41598-018-33254-9)
Supplement: Supplementary file 1 — Supplementary Material [file 41598_2018_33254_MOESM1_ESM.docx]

***Ex vivo* assessment and *in vivo* validation of non-invasive stent monitoring techniques based on microwave spectrometry**

Carolina Gálvez-Montón, DVM, PhD,^1,2#^ Gianluca Arauz-Garofalo,^3#^ Oriol Rodriguez-Leor, MD, PhD,^2,4,5#^ Carolina Soler-Botija, PhD,^1,2^ Susana Amorós García de Valdecasas,^6^ Flavio David Gerez-Britos,^3^ Antoni Bayes-Genis, MD, PhD, FESC,^1,2,4,5^ Juan Manuel O’Callaghan, PhD,^6^ Ferran Macià, PhD,^3^ Javier Tejada, PhD,^3^

^1^ICREC Research Program, Fundació Institut d'Investigació en Ciències de la Salut Germans Trias i Pujol, Barcelona, Spain.

^b^CIBER Cardiovascular, Instituto de Salud Carlos III, Madrid, Spain.

^c^Grup de Magnetisme, Departament de Física de la Matèria Condensada, Universitat de Barcelona, Barcelona, Spain.

^d^Servei de Cardiologia. Hospital Universitari Germans Trias i Pujol, Badalona, Spain.

^e^Departament de Medicina, Universitat Autònoma de Barcelona, Barcelona, Spain.

^f^ CommSensLab, , Universitat Politècnica de Catalunya, Barcelona, Spain.

**SUPPLEMENTARY MATERIAL**

**METHODS**

**In vitro microwave spectrometry setup**

MWS is based on the analysis of the interaction between an incident electromagnetic wave and high conductivity material such as a metallic stent. Depending on the frequency and orientation of the incident wave, stents exhibit characteristic behaviors from which we can obtain information about their geometry and environment. Our MWS setup works by sweeping the frequency $(f)$ and incidence angle of the wave $\left( \varphi\right)$. As a result, and as detailed below, the entire characterization of a given stent can be encapsulated in a two-dimensional chart that can be analyzed at a glance.

Supplementary Figure 1 shows the MWS apparatus used in the present study. The components enabling the frequency sweep of the microwave fields are the backbone of this setup. They consist of a couple of JXTXLX-20180 antennas (Chengdu A-info Inc., Chengdu, China) connected to a HP 8510C network analyzer (Agilent Technologies, Santa Clara, CA, USA) via 3GW40-0TD01D02048.0 coaxial feedlines (W.L. Gore & Associates Inc., Newark, DE). Therefore, by placing a stent in the midpoint between the antennas we acquire the transmission coefficient ($S\left( f,\varphi\right)$) between the transmitting and receiving antenna at each frequency $(f)$ and angle $\left( \varphi\right)$. We then normalize $S\left( f,\varphi\right)$ with respect to the transmission coefficient of the setup without the stent $S_{ref}\left( f \right)$ to obtain the absorbance $A\left( f,\varphi\right)$:

$$A\left( f,\varphi\right)=\left| \frac{S\left( f,\varphi\right)}{S_{ref}\left( f \right)} \right|$$

$A\left( f,\varphi\right)$ may be calculated in decibels (dB) : $A\left( f,\varphi\right)_{dB}=20 logA\left( f,\varphi\right)$ By using absorbance we reduce the effect of reflections and scattering of the microwave signal by nearby objects.

Sample positioning was achieved with the aid of an expanded polystyrene column mounted on a ST2818M1006-B stepping motor (Nanotec Electronic GmbH & Co. KG, Munich, Germany). This column-motor assemblage works as a rotating platform that allows scanning of the stent around the azimuthal angle, *ϕ*, formed by the stent axis and the line joining the antennas. The frequency sweep runs between 1.50 and 17.50 GHz in steps of 0.02 GHz, and the angle scan is from 0.0° to 360° in steps of 1.8°. Thus, the absorbance as a function of *f* and *ϕ*, *A*(*f*,*ϕ*), is recorded. Finally, the entire *A*(*f*,*ϕ*) dataset can be represented in a single chart using graphing software.

**In vitro stent testing**

For the fracture test we chose a Medtronic Endeavor Sprint DES with nominal length *ℓ*_nom_ = 30 mm and nominal diameter *d*_nom_ = 2.50 mm. For the recoil test we used a Medtronic Endeavor Resolute DES with *ℓ*_nom_ = 15 mm and *d*_nom_ = 3.00 mm. Finally, for the in-stent lipid occlusion test, we used the Medtronic Driver Sprint BMS with *ℓ*_nom_ = 12 mm and *d*_nom_ = 2.75 mm.

The stents used in the fracture test and in-stent lipid occlusion test were originally expanded up to their nominal inflation pressures (*p*_nom_ = 0.91 ± 0.05 MPa) in order to obtain their corresponding nominal sizes. We used a compatible inflation device, the Encore 26 Advantage Kit (Boston Scientific Corp., Natick, MA, USA), to expand the Rapid Exchange Stent Delivery System balloon catheter (Medtronic, Inc., Minneapolis, MN, USA) on which these stents were mounted. The stent used in the recoil test followed a different process as explained below.

Stent fracture test

After completing the expansion process, the intact stent was labeled segment AB, and its real length, *ℓ*_AB_, was measured using an IP67 digital caliper (Vogel Germany GmbH & Co., Kevelaer, Nordrhein-Westfalen, Germany). Its *A*(*f*,*ϕ*) chart was acquired, and the intact state of segment AB characterized. At this point, we practiced a single cut on the weld linking crowns #15 and #16 with customized precision scissors, which led to a complete transverse linear stent rupture classified as a grade III fracture (1,2). The A and B resulting segments and their real lengths, *ℓ*_A_ and *ℓ*_B_, were measured. The *A*(*f*,*ϕ*) chart for the fractured stent was acquired by placing both segments together on the sample holder with a 1 mm gap in between. For completion, we also acquired the *A*(*f*,*ϕ*) chart for each segment separately.

Stent recoil test

To quantify the diametrical shrinkage of the stent, we defined the recoil degree as a function of the stent diameter as follows:

$$r(d)=\frac{d_{M}-d}{d_{M}-d_{m}}$$

, where, *d*_m_ and *d*_M_ are respectively the minimum and maximum stent diameter values arbitrarily selected to define *r*. For *d*_m_ we chose the deployment diameter at *p* = 0.0 MPa, which corresponded to the diameter measured just after unpacking the stent from its commercial kit (*d*_m_ = 1.190 ± 0.015 mm). In this state, the stent was completely cramped on the balloon catheter on which it was mounted, and the corresponding recoil degree was maximized (*r*(*d*_m_) = 1). For *d*_M_ we took the deployment diameter at *p* = 2.33 ± 0.05 MPa, which was 3.964 ± 0.013 mm. At that point, the recoil degree was minimized (*r*(*d*_M_) = 0).

In-stent lipid occlusion test

This protocol was separated into three phases. First, we acquired the *A*(*f*,*ϕ*) chart for the stent just after completing its expansion. We also measured its mass with a MI 220 CBC scale (Cobos Precision SL, Barcelona, Spain), its length, and its diameter. The cholesterol deposition process began in the second stage by spraying the stent with a cholesterol-saturated (94%, Sigma-Aldrich) ethanol (96%, Panreac) solution. The resulting dilution-drenched stent was submitted to a drying air flux to accelerate the solute deposition. Complete evaporation of the solvent left a few milligrams of cholesterol on the stent wires, which eventually filled the space between adjacent crowns. The total cholesterol mass left on the stent, *m*, was determined by subtracting the mass of the stent recorded in the initial stage. After *m* was measured, the *A*(*f*,*ϕ*) chart was acquired for the stent-cholesterol sample. This entire protocol was iterated until a thick cylindrical shell of cholesterol formed around the stent, providing a series of *A*(*f*,*ϕ*) charts for increasing *m* values. Finally, in the third stage, we removed the entire cholesterol crust deposited on the stent by repeatedly washing it in ethanol at 60°C. When the stent was completely cleaned, we acquired its *A*(*f*,*ϕ*) chart and measured its mass and dimensions as described in the first stage.

**In vivo microwave spectrometry setup**

It consisted of a two-port probe connected to a vector network analyzer (E8361A, Keysight Technologies) via coaxial feed lines (3GW40-0TD01D02048.0, W. L. Gore & Associates) (Figure 1B). The probe was used to launch a microwave electromagnetic excitation onto the stent and to capture its response. The transmission spectrum (*TS*) was acquired at a frequency range of 0.1 to 6.0 GHz, to identify the resonance frequencies characteristic of a stent implanted in mice.

Measurements were performed with anesthetized mice lying in a prone position on a padded surface, and their backs were palpated with the MWS probe. Ultrasound transmission gel (Aquasonic 100, Parker Laboratories, Inc.) was applied on the probe head to soften the medium transitions and convey microwaves without crossing air. Each experimental mouse characterization comprised three measurements at different scanning zones. The first two zones were both with the probe centered above the stent, one parallel and the other angled at 30º with respect to the stent axis. The third zone was shifted 10 mm to the right of the paravertebral position. Sham mouse characterizations comprised 3 measurements at the same scanning zones, but they captured tissues that received a simulated stent implantation.

**Quantitative analysis of the stent resonance frequency**

It is well known that the acoustic frequency emitted by a resonating tuning fork depends on whether its surrounding medium is one or another. Similarly, the microwave resonance frequency (*f)* of a stent may shift when its surrounding medium is changed. The physical property behind such electromagnetic frequency shift is the relative permittivity of the medium (*ε_r_*) (1,2). The general equation linking resonant frequency of a stent inside two non-magnetic media A and B is (3-5):

$$f_{B}=f_{A}\sqrt{\frac{\varepsilon_{r}^{A}}{\varepsilon_{r}^{B}}}$$

(Equation 1)

where *f*_A_ and *f*_B_ are the stent resonant frequencies in media A and B, respectively, and *ε*_r_^A^ and *ε*_r_^B^ are their corresponding relative permittivities. Particularizing Equation 1 for air and mice connective tissue as media A and B we reach:

$$f_{\mathrm{mice}}=f_{\mathrm{air}}\sqrt{\frac{\varepsilon_{r}^{\mathrm{air}}}{\varepsilon_{r}^{\mathrm{mice}}}}$$

(Equation 2)

**REFERENCES**

1. Pozar DM, Microwave Engineering, 4th edition, John Wiley & Sons, New York, 2012.

2. Balanis CA, Antenna Theory: Analysis and Design, 3rd edition, John Wiley & Sons, New York, 2005.

3. Arauz-Garofalo G, López-Domínguez V, Hernàndez JM, Rodríguez-Leor O, Bayés-Genís A, O'Callaghan JM, García-Santiago A, Tejada J. Microwave spectrometry for the evaluation of the structural integrity of metallic stents. Med Phys 2014;41:041902-13.

4. Arauz-Garofalo G, López-Domínguez V, Hernàndez JM, Rodriguez-Leor O, Bayes-Genis A, Rius JM, O’Callaghan JM, García-Santiago A, Tejada J. RF monitoring of commercial vascular stents with dipole scattering resonances. In IEEE International Microwave Symposium. "2014 IEEE MTT-S International Microwave Symposium (IMS 2014): Tampa, Florida, USA: 1-6 June 2014". Tampa, FL: Institute of Electrical and Electronics Engineers (IEEE), 2014, p. 1-4.

5. Arauz-Garofalo G, Camacho-Puerma LM, García-Santiago A, Tejada J. (2015). Microwave spectrometry for the evaluation of in-stent neoatherosclerosis. Biomedical Physics & Engineering Express, 1(3), 035202.

**SUPPLEMENTARY FIGURE LEGENDS**

**Supplementary Figure 1**. Sketch of the setup used to obtain MWS absorbance charts for stents. Port 1 of the network analyzer (A) sends a stimulus to the emitter antenna (B) via a coaxial feedline (C). The generated MW signal travels through free space until interacting with the stent. The emerging signal is collected by the receiver antenna (D) and sent back to port 2 of the network analyzer (E) via the second feedline (F). The PC (G) connected to the network analyzer records the frequency scan of the stent in the 1.50–17.50 GHz range. This PC also controls the stepper motor (H) using a single board microcontroller (I). Finally, the stepper rotates the sample holder (J) on which the stent lies, enabling its 0–360° angular scan. The inset shows the rotation axis (vertical) and the azimuthal angle, *φ*, established by the stent axis and the antenna axis (both lying in the plane perpendicular to the rotation axis).

**Supplementary Figure 2.** Dielectric permittivity of mice. The black solid line represents the experimental dielectric permittivity as a function of the microwave frequency, εmice(f), measured with Dielectric Probe Kit 87050C (Keysight Technologies, Santa Clara CA). The grey-shaded region highlights the error band of the experimental value. Black dotted lines denote the relative permittivity of mice tissue at f = fair = (5.071 ± 0.014) GHz, which is εmice = 41 ± 3

**SUPPLEMENTARY FIGURES
Supplementary Figure 1**

**
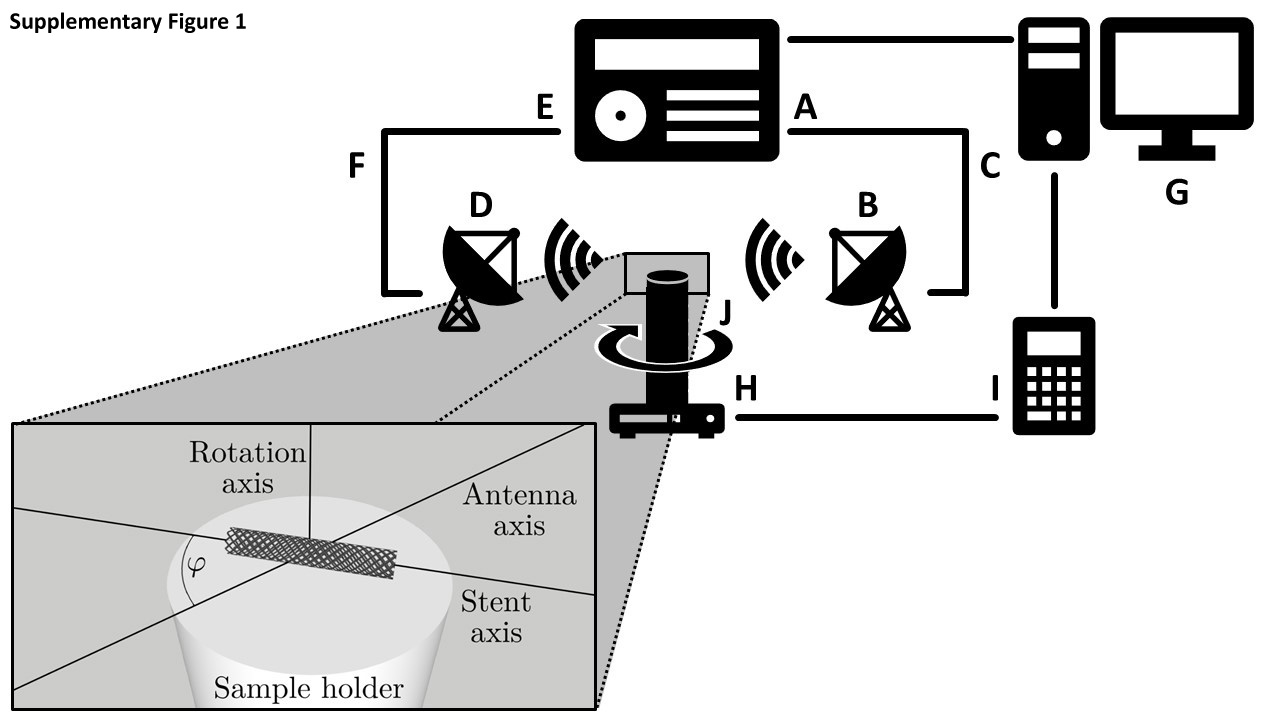
**

**Supplementary Figure 2**

**
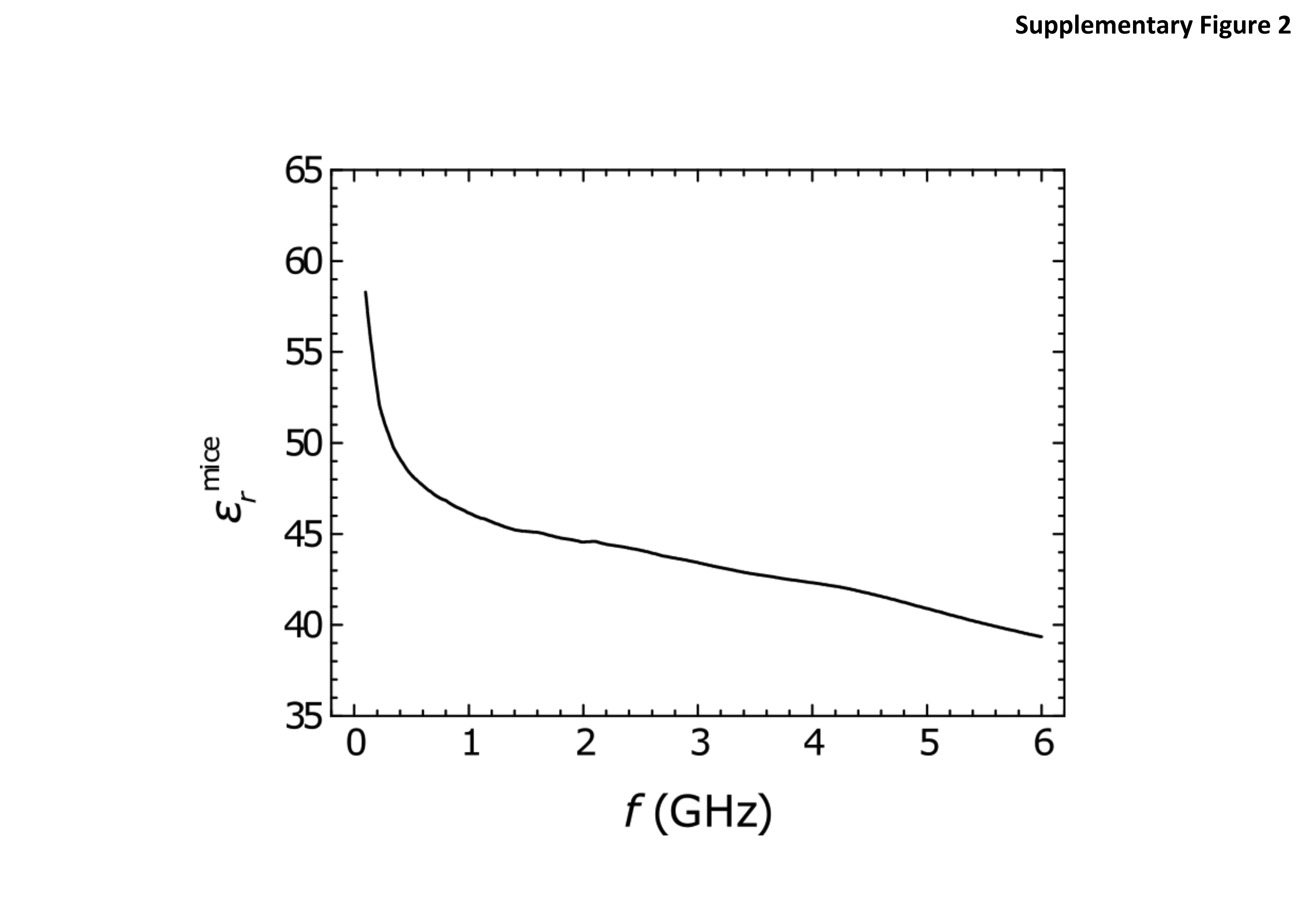
**

**SUPPLEMENTARY TABLES**

**Supplementary Table 1**. Summary of the three Medtronic stents used in the *ex vivo* study.

| **Model** | **Type** | ***ℓ*_nom_**  **(mm)** | ***d*_nom_**  **(mm)** | **Crowns** | **Crown tips** | **Welding pattern** |
| --- | --- | --- | --- | --- | --- | --- |
| Endeavor Sprint* | DES | 30 | 2.50 | 25 | 7 | Single helix |
| Endeavor Resolute* | DES | 15 | 3.00 | 15 | 10 | Double helix |
| Driver Sprint* | BMS | 12 | 2.75 | 10 | 7 | Single helix |

*These stents share the same nominal inflation pressure (*p*_nom_) of 0.91 ± 0.05 MPa and the same architecture with characteristic rhomboid cells. Their sinusoidal modular elements (crowns) are made with a 0.091-mm-thick round wire of F562 cobalt-chromium alloy, which are held together by a helical laser-fused welding pattern. The number of crowns and number of tips on each crown varied according to the nominal length (*ℓ*_nom_) and the nominal diameter (*d*_nom_) of each stent. DES = Drug-eluting stent; BMS = Bare-metal stent.
